# Clinical anatomy of the cephalic vein for safe performance of venipuncture

**DOI:** 10.1186/s40981-017-0121-6

**Published:** 2017-09-11

**Authors:** Mitsuhiro Matsuo, Satoru Honma, Takahiro Sonomura, Mitsuaki Yamazaki

**Affiliations:** 1 Department of Anesthesiology, Faculty of Medicine, University of Toyama, 2630 Sugitani, Toyama, 930-0194 Japan; 2 Anatomy II, Kanazawa Medical University, Uchinada, Japan

**Keywords:** Cephalic vein, Superficial branch of the radial nerve, Venipuncture, Nerve injury

## Abstract

**Background:**

The aims of this study were to elucidate why the cephalic vein provides a reliable cannulation site from a morphological viewpoint and identify an effective landmark for avoiding injury to the superficial branch of the radial nerve (SBRN), allowing for safe venipuncture of the cephalic vein.

**Findings:**

We examined 32 forearms and wrists from 18 cadavers. The cephalic vein was a constant structure containing a branch communicating with a collateral vein of the deep palmar arch via the first dorsal interossei muscle. The metacarpal vein from the medial two digits flowed into the cephalic vein. The venous confluence formed 5.8 ± 1.2 cm proximal to the radial styloid process. The SBRN passed 0.4 ± 0.3 cm volar to the venous confluence. The distance between the venous confluence and subcutaneous emergence of the SBRN was 2.6 ± 1.0 cm.

**Conclusions:**

These observations suggest that the cephalic vein is a constant structure that serves as a drainage vein of the hand and provides a reliable cannulation site in the forearm. The venous confluence may serve as a novel landmark to predict the running course of the SBRN.

## Findings

### Introduction

The cephalic vein, which passes the anatomical snuff box in the wrist, is one of the most frequently selected sites in the distal forearm for venous cannulation in Japan [1]. The superficial branch of the radial nerve (SBRN), which runs close to the cephalic vein, exhibits various bifurcation patterns [2, 3]. The point at which the cephalic vein crosses the SBRN is highly variable [4]. Therefore, there is a risk of nerve injury when puncturing the cephalic vein and subsequent development of causalgia or neuromas [5, 6]. Although the cephalic vein should not be selected for the first puncture attempt, this vein can provide a reliable cannulation site when it is difficult to obtain other venous access [7]. In this study, we performed cadaveric dissection to elucidate why the cephalic vein provides a reliable cannulation site from a morphological viewpoint and identify an effective landmark with which to avoid injury to the SBRN, thus ensuring safe venipuncture of the cephalic vein.

### Materials and methods

**Fig. 1:**
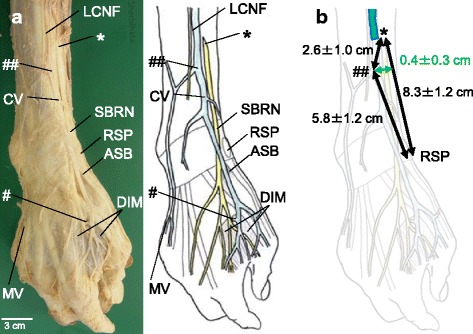
Gross anatomy of the cephalic vein and superficial branch of the radial nerve (SBRN). **a** Macroscopic appearance of the forearm. **b** Distance between radial styloid process (RSP) (* to ##) is shown as mean ± standard deviation. Blue area shows a suggestive safe area for cannulation to the CV. ASB anatomical snuff box, CV cephalic vein, DIM first dorsal interossei muscle, LCNF lateral cutaneous nerve of the forearm, MV metacarpal vein. ^#^Communicating branch of CV to deep palmar vein. ^##^Confluence of CV and metacarpal vein. *Emergence of SBRN

This study was approved by the Ethical Committee of Kanazawa Medical University (no. E211). We examined 32 forearms and wrists from 18 cadavers (12 males, 6 females) with no known trauma or disease in the upper limbs. All cadavers had been donated to Kanazawa Medical University for medical education. The mean age of the cadavers was 80 years. Distance measurements were made (Fig. 1), and the data are shown as the mean ± standard deviation of 31 forearms.

### Results

The cephalic vein, which ran over the anatomical snuff box, was identified in all arms. As shown in Fig. 1, each cephalic vein had a branch that communicated with a collateral vein of the deep palmar arch via the first dorsal interosseous muscle in 32 of 32 cases (100%). Although cutaneous veins are generally derived only from the skin, the cephalic vein is also derived from the hand. In contrast to the cephalic vein, no cutaneous veins in the present study had branches that directly connected to the deep palmar vein. The SBRN emerged from under the brachioradialis and coursed between the tendons of the extensor carpi radialis longus and the brachioradialis in 31 of 32 cases (97%). In one arm, the SBRN was lacking, and the lateral cutaneous nerve of the forearm derived from the musculocutaneous nerve compensated for its absence. The SBRN emerged at a mean distance of 8.3 ± 1.2 cm (range, 5.5–10.4 cm) proximal to the radial styloid process. The metacarpal vein from the medial two digits flowed into the cephalic vein. The venous confluence formed 5.8 ± 1.2 cm (range, 4.1–7.8 cm) proximal to the radial styloid process. The SBRN passed 0.4 ± 0.3 cm (range, 0.0–1.0 cm) volar to the venous confluence. The distance between the venous confluence and subcutaneous emergence of the SBRN was 2.6 ± 1.0 cm (range, 0.7–4.6 cm). The SBRN ran beside the cephalic vein in the distal forearm and crossed the inferior aspect of the cephalic vein in all arms that possessed the SBRN.

### Discussion

In this study, we revealed that the cephalic vein is consistently derived from a collateral vein of the deep palmar arch. These observations suggest that the cephalic vein exists constantly because of a drainage vein of the hand. Interestingly, the communicating branch is illustrated in anatomy textbooks such as *Grant’s Atlas of Anatomy* [8] and *Toldt-Hochstetter Anatomischer Atlas* [9], but it is not documented in detail. Unlike other unnamed cutaneous veins which show a variety of running courses, the cephalic vein constantly exists and seems to provide a reliable cannulation site in the distal forearm.

The point at which the SBRN emerges in the subcutaneous fat was at a mean of 8.3 cm from the radial styloid process in this study using Japanese cadavers, which is consistent with previous studies [2–4]. It is likely that the distance from emergence of the SBRN to the radial styloid process is not affected by sex or ethnicity [10]. In addition to the radial styloid process, the dorsal tubercle of the radius is another bony landmark. Robson et al. [2] reported that the SBRN passes 1.49 cm radial to the dorsal tubercle of the radius. In the present study, the venous landmark (confluence of the cephalic vein from the ulnar-side metacarpal vein) was 2.6 cm (range, 0.7–4.6 cm) distal to the emergence of the SBRN and 0.4 cm volar to passing the nerve. In other words, the cephalic vein at a point ≥ 4.6 cm proximal to the venous confluence should be selected as the cannulation site to avoid injury to the SBRN. Consistent with these observations, the figures in previous reports [2, 4, 11] show that the SBRN passes just under or volar to the venous confluence. Further studies are needed to confirm the availability of the venous confluence as a visible landmark for cephalic vein cannulation.

The SBRN ran beside the cephalic vein in the distal forearm and crossed the inferior aspect of the cephalic vein in this study, which is consistent with a previous investigation [4]. Vialle et al. also have showed that the crossing point ranged from 9 cm proximally from the radial styloid process [4]. In case the cephalic vein is selected as the venous cannulation site, penetration of the posterior wall of the cephalic vein must be avoided. Additionally, as noted in case series describing iatrogenic SBRN injury [6], the use of a 22-gauge or smaller needle might be helpful to avoid significant nerve injury. The lateral cutaneous nerve of the forearm, the diameter of which is smaller than that of the SBRN, often runs over the cephalic vein (Fig. 1). Although no reports have described lateral antebrachial cutaneous nerve injury in the distal forearm, the nerve can also be injured following cephalic vein cannulation.

Besides nerve injury, arterial injury is also reported a less common complication when puncturing the cephalic vein [12, 13]. The arterial injury may occur only when the superficial radial artery is present. The superficial radial artery is an anatomic variation of the radial artery which running over the anatomical snuffbox, and its frequency has been reported as 0.5–1% [12].

### Conclusion

In summary, this study addressed the morphological reason why the cephalic vein provides a reliable cannulation site and identified a superficial landmark with which to avoid SBRN injury. There is a substantial risk of SBRN nerve injury when puncturing the cephalic vein. In case the cephalic vein is selected as the venous cannulation site, the cephalic vein at a point ≥ 4.6 cm proximal to the venous confluence should be selected as the cannulation site to avoid injury to the SBRN. Although a limitation of this study is the small number of cadavers in the dissection series, these findings may contribute to safe venipuncture of the cephalic vein.
